# “I had eyes on me to help me”: a qualitative descriptive study on trust in passive monitoring systems among older adults with mild cognitive impairment

**DOI:** 10.1093/gerona/glag018

**Published:** 2026-01-30

**Authors:** Oonjee Oh, Sean Harrison, Justine S Sefcik, Therese S Richmond, Nancy Hodgson, George Demiris

**Affiliations:** School of Nursing, University of Pennsylvania, Philadelphia, Pennsylvania, United States; School of Nursing, University of Pennsylvania, Philadelphia, Pennsylvania, United States; College of Nursing and Health Professionals, Drexel University, Philadelphia, Pennsylvania, United States; School of Nursing, University of Pennsylvania, Philadelphia, Pennsylvania, United States; Penn Injury Science Center, University of Pennsylvania, Philadelphia, Pennsylvania, United States; School of Nursing, University of Pennsylvania, Philadelphia, Pennsylvania, United States; School of Nursing, University of Pennsylvania, Philadelphia, Pennsylvania, United States; (Medical Sciences Section)

**Keywords:** Depth sensors, Machine learning, Trust in technology, Fall prevention

## Abstract

**Background:**

Passive monitoring can support aging in place for older adults with mild cognitive impairment (MCI). Yet, their trust in the technology is crucial as the devices are installed in their homes, collecting data throughout their daily activities. We aimed to understand the perceptions of older adults with MCI to having passive monitoring technology embedded in their residence and examine their trust within this context.

**Methods:**

For this qualitative descriptive study, data were obtained from a 12-month study that used passive monitoring technology to predict fall risks among community-dwelling older adults with MCI. We analyzed 30 exit interviews via directed content analysis using trust-related constructs outlined by Lankton et al.

**Results:**

Findings are presented under the model’s constructs. Under reliability and integrity, participants described their perception of the sensor’s omnipresence, its impact on privacy, and interactions with the team that made them feel safe. Under helpfulness and benevolence, we identified participants’ views on the device’s benefits, as well as the importance of having someone who is responsive to their data. Under functionality and competency, participants reflected on the sensor’s intended unobtrusiveness and its accuracy in capturing gait patterns, along with the necessary infrastructures and indicators for proper functioning.

**Conclusions:**

Participants conceptualized depth sensors as more than a device, instead associating it with human agents who monitored them and interpreted their data. Despite the passive nature of the technology, successful implementation of monitoring systems requires ongoing human involvement and communication with the participants to build trust within the community.

## Introduction

Among older adults, falls are very common, but they can have serious physical consequences, such as hip fractures, head bruises, soft tissue injuries, and morbidity or death.^1^ Approximately 29% of noninstitutionalized older adults in the United States reported falling at least one time a year,^2^ with estimated medical expenses due to falls reaching around $50 billion.^3^ In the older adult population, those with mild cognitive impairments (MCI) represent a unique group who maintain independence in basic activities of daily living but experience a change in cognitive abilities (eg, memory, executive functions, language, or visuospatial abilities),^4^ which are crucial for safe mobility. In a recent meta-analysis, Hopkins et al.^5^ reported that the fall prevalence of older adults with MCI ranged from 34% to 46%, depending on the data collection method and assessment period. This study confirmed that, among this population, slow gait speed, poor postural control, non-amnestic MCI, and dual tasks performance (ie, walking while doing another task) were significant risk factors for falls.^5^ However, evidence-based interventions to prevent fall risks are still lacking for older adults with MCI. Choi et al.^6^ identified only 17 articles on fall prevention interventions targeting cognitively impaired older adults, but their review encompassed studies on dementia as well. Zeng et al.^7^ identified 32 randomized clinical trials that tested the effectiveness of exercise on mobility, postural control, and falls among older adults with MCI, but could not conduct a meta-analysis on fall-related outcomes due the limited number of studies and sample size.

Passive sensing and machine learning (ML) are promising methods to identify fall risks in community-dwelling older adults with MCI.^8^ Passive sensors embedded within homes provide an omnipresent monitoring of the individuals’ motions without requiring participants to learn how to use a device, thereby reducing the time and cognitive burden on them.^9^ Moreover, as sensors collect real-time data on older adults’ gait and functional mobility at home, personalized ML models can capture clinically meaningful changes that serve as early warning signs of fall risk. Such signals of deterioration can inform timely health interventions to prevent falls and support independent living of older adults with MCI.

In a previous mapping review, Schroeder et al.^10^ identified trust in the system as a factor associated with older adults’ intention to adopt new technology. Trust in technology is defined as the end users’ willingness to depend on the technology for certain tasks that may involve uncertainty or risk, based on the belief that it possesses positive and desirable attributes.^11^ Trust is a crucial concept in passive monitoring because the technology requires depth sensors to be installed in older adults’ personal residence and constantly collects data throughout daily activities. Read et al.^12^ reported that, while passive monitoring systems help support older adults’ aging in place, having sensors in households may be perceived as invasive by some, acting as a barrier to the systems’ implementation within the community. In our recent study exploring the feasibility of a fall risk prevention intervention incorporating passive ambient sensor technology,^13^ several participants commented that they trusted the research team who introduced the technology. However, because the intervention also included biweekly coaching sessions, attributes of the depth sensor itself that made it trustworthy were not specifically explored. Lie et al.^14^ also explored older adults’ trust in home monitoring systems, but the technology in their study was aimed at community-dwelling older adults without health problems and focused on the broad detection of unusual changes in daily activities. Hence, research is needed to explore what makes passive in-home sensing systems trustworthy for older adults with MCI in predicting fall risk, to ensure that such technologies are designed in ways that promote user acceptability.

Within the technology literature, two different constructs of technology have been used to explore trust: system-related trust beliefs, where technology is viewed as an artificial system, and human-like trust beliefs, where technology is perceived as if it were human.^15^ Earlier theoretical foundations of trust in technology were based on interpersonal or interorganizational models; hence, the focus was often geared towards the trust in humans or organizations providing the technology.^16^ One sentinel paper underlying such interpersonal trust belief is by Mayer et al.,^17^ who identified three human-like constructs explaining trustworthiness: ability, benevolence, and integrity. Over time, other perspectives emerged, where researchers viewed the artificial system itself as the object of trust.^18^ For instance, McKnight et al.^19^ explored what technology attributes make it trustworthy, irrespective of the humans or organization overseeing it, and identified three system-like trusting beliefs: functionality, helpfulness, and reliability. Both technology constructs have been widely used in empirical studies, and Lankton et al.^15^ synthesized pairs of trust-related constructs, mapping human-like trust beliefs to their corresponding system-like trust beliefs: (1) integrity and reliability, (2) benevolence and helpfulness, and (3) competence and functionality.

In terms of passive monitoring, both system- and human-like trust beliefs could be applied: While older adults may view depth sensors as devices that keep them safe in their home environment, they may also associate them with external agents monitoring them, impacting their privacy.^14^ However, as there have been no prior studies focusing on how older adults with MCI perceive this technology for fall risk identification, it is unknown how trust-related constructs apply in this context. Closing this knowledge gap is crucial to ensure that tools developed to support aging in place are driven not only by technological possibilities but also by the acceptability and needs of the primary users.^20^ Such a user-centric perspective aligns with the growing recognition that end users should be seen as active partners who inform the design and implementation of technology rather than mere recipients,^21^ and it provides a strong foundation for future user-centered or participatory design approaches. Therefore, we aimed to describe how community-dwelling older adults with MCI perceive and trust passive monitoring in their residence. Using paired constructs of trust beliefs outlined by Lankton et al.,^15^ we captured trust-related narratives from older adults with MCI who had one year of experience with passive sensing devices in their homes.

## Methods

### Study design

For this qualitative descriptive study, data were obtained from a 12-month study examining the feasibility of a passive in-home sensing system using ML to identify early signals for fall risk among older adults with MCI living in the community.^8^ We conducted a directed content analysis^22^ to validate or expand existing descriptions of trust-related constructs outlined by Lankton et al.^15^ in the context of passive monitoring targeting community-dwelling older adults with MCI. We adhered to the COREQ (consolidated criteria for reporting qualitative studies) guidelines (Table S1).^23^

### Setting and procedures

For recruitment, we utilized convenience sampling and conducted in-person informational sessions at living communities and community centers in Philadelphia, PA, USA. Follow-up meetings were conducted with older adults who expressed interest, during which screening for study eligibility was conducted. Inclusion criteria included (1) age 65 years or older, (2) currently residing in a senior living community with internet connection, (3) able to walk in household distances, typically ranging from 10 to 30 m, as assessed by the research team member, and (4) complete timed-up-and-go assessment,^24^ score ≥17 on the Montreal Cognitive Assessment,^25^ and score ≥28 (education adjusted) on the Telephone-Based Neurocognitive Screening Instrument.^26^ Older adults were excluded if they had plans to move, were reliant on wheelchairs or scooters within their households, or were diagnosed with a terminal illness, central nervous system disorders (eg, stroke or Parkinson’s disease), dementia/Alzheimer’s disease, or serious vision or hearing deficits despite corrections (eg, glasses, hearing aids) that would affect their gait. If eligible, informed consent was obtained and participants were educated about the device and its functionality. Staff from the older adult communities or buildings did not receive training on the device and did not engage in any study activities.

One commercially available depth sensor (Foresite HealthCare) was installed in the living room of each apartment (Figure 1), and the in-home sensor system continuously monitored the older adults’ movement within the sensor range. The device used infrared depth sensing and transmitted raw data to secure cloud storage, from which gait parameters were derived and a silhouette image of the participant was generated (Figure 2). The extracted silhouetted depth images were dual-purposed to ensure privacy and to quantify gait data. From the silhouettes, a pre-designed algorithm was used to detect and measure participants’ walks, extract gait-related parameters, and aggregate these metrics into daily gait summaries. The system established a personalized model for each person by entering a calibration period of up to 10 days starting from the installation date, during which all walks within the device’s range were recorded. After the calibration period, demographics (ie, participants’ height, age, use of walking aids [e.g., cane, walker]) were entered, along with attestation of the participants’ walking pattern (ie, ≥2 m, ≥5 times/day). The model was not updated during the study period and participants’ gait data were displayed onto a user-friendly dashboard, where notable changes in gait parameters created system alerts.

**Figure 1.**
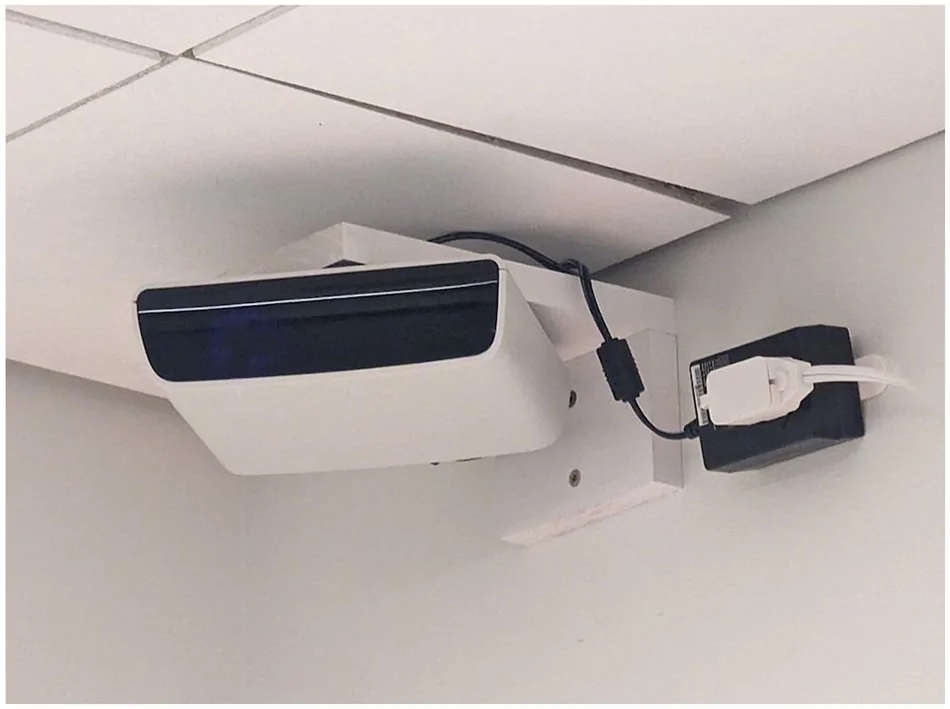
Image of the depth sensor.

**Figure 2.**
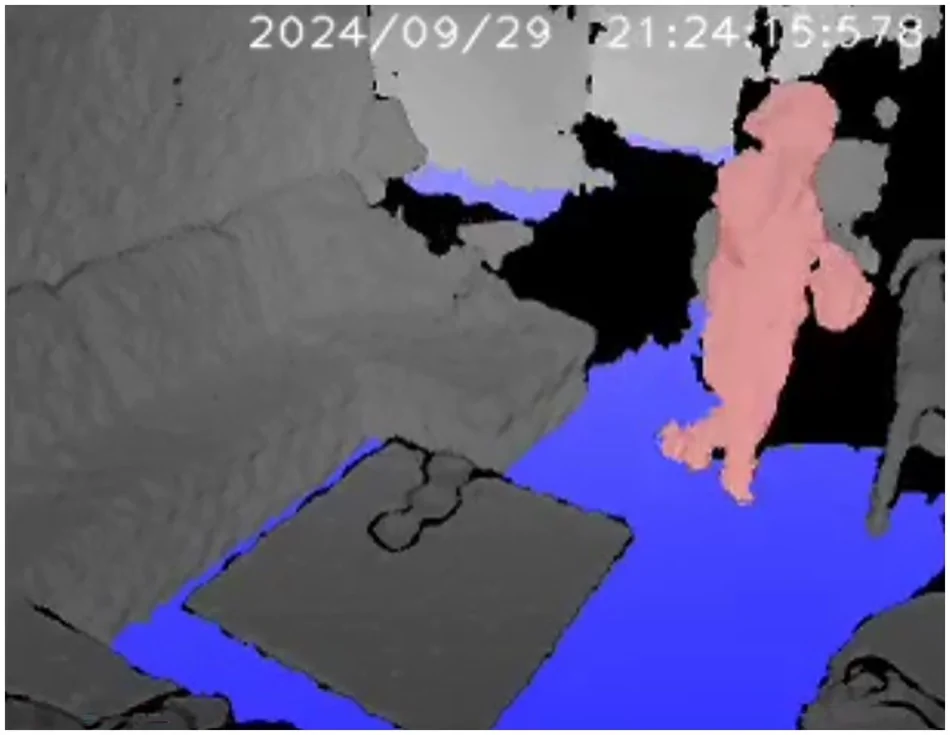
Image of the silhouette extraction created by the depth sensor.

Throughout the study year, we made quarterly home visits (weeks 12, 24, 36, 48), with telephone interviews interspersed (weeks 6, 18, 30, 42), to collect information about their physical and cognitive performances and daily activities. Adverse events (eg, recent falls, near falls, hospitalization, illness, or unexpected doctor visits) were self-reported by participants every 6 weeks. The Institutional Review Board of the University of Pennsylvania approved the study’s protocol (02/08/2021, #849231).

A total of 78 older adults with MCI enrolled, of whom 67 completed the entirety of the study. Two expired and nine withdrew due to various reasons (eg, installation, internet, or other technical issues, physical inactivity, and personal reasons unrelated to the study). With those who completed the study, the second author (SH) conducted individual exit interviews in their residences, using a semistructured interview guide. Questions were designed based on the conceptual framework of obtrusiveness in home-based monitoring technologies, which defines obtrusiveness as the degree to which end users perceive passive monitoring technology as physically or cognitively prominent.^27^ Participants were asked about their overall experience with the sensor, the impact the sensor had on their daily routines, home environment, personal space, interactions with others, and sense of independence, as well as its perceived usefulness and privacy concerns (Table S2). During the interviews, participants were also shown their data and dashboard displays and were then asked additional questions, such as their general thoughts on the display, its perceived accuracy, and their willingness to share the data with others. To examine the clarity and comprehension of the questions, the interview guide was pilot-tested with five older adults prior to the study. Interviews lasted approximately 30 min and were all audio-recorded. Field notes were not documented during the interview and no repeat interviews were carried out.

### Data analysis

The dataset consisted of interviews from a sample of 30 participants randomly selected from the total pool of 67 who completed the study. Interviews were transcribed verbatim and uploaded to the Atlas.ti software for data management and analysis.^28^ Transcripts were not returned to the participants.

Using the trust constructs from Lankton et al.^15^ as predetermined categories, deductive coding was independently conducted by two authors (OO and GD) on 10 transcripts, followed by discussions until reaching consensus. Subsequently, the first author (OO) coded the remaining 20 transcripts. After coding 30 transcripts in total, three additional randomly selected transcripts were assessed to confirm data redundancy.^29^ New codes or ideas did not emerge from these additional transcripts, confirming that data redundancy was achieved.

Within each predetermined category, the first author examined the existence of a hierarchical structure and inductively identified subcategories. Two authors (OO and GD) had regular meetings to discuss findings. In addition to investigator triangulation, trustworthiness was ensured by employing peer debriefing where the entire team reviewed the findings and the written work for critical feedback,^30^ the research team’s prolonged engagement in the field throughout the 12-month study period, and an audit trail that documented all analytic decisions.^31^ Details regarding the study team’s reflexivity is available (Table S3).

## Results

A total of 30 older adults with MCI were included in this analysis (Table 1). The sample’s age ranged from 65 to 88, with a mean age of 72.7 (standard deviation = 6.0). Most were female (86.7%) and identified as Black or African American (75.9%). Approximately half reported having some college education (56.7%). One third of the sample was dually enrolled in Medicare and Medicaid (30.0%). All participants were living alone, with slightly less than half using walking aids (43.3%).

**Table 1.** Sample characteristics (*N* = 30).

Variable	Mean (standard deviation) or *n* (%)
**Age**	72.7 (6.0)
**Gender**	
** Female**	26 (86.7)
** Male**	4 (13.3)
**Education**	
** Less than high school**	1 (3.3)
** High school diploma/GED**	7 (23.3)
** Some college**	17 (56.7)
** Bachelors**	3 (10.0)
** Graduate/Professional^a^**	2 (6.7)
**Ethnicity**	
** Not Hispanic/Latino**	30 (100.0)
** Hispanic/Latino**	0 (0.0)
**Race**	
** Black/African American**	22 (75.9)
** White**	5 (17.2)
** American Indian/Alaska Native**	0 (0.0)
** Asian/Asian American**	0 (0.0)
** Native Hawaiian/Pacific Islander**	0 (0.0)
** More than one race**	2 (6.9)
**Marital status**	
** Divorced**	14 (46.7)
** Single**	9 (30.0)
** Widowed**	6 (20.0)
** Married/Partnered**	0 (0.0)
** Other**	1 (3.3)
**Living alone**	
** Yes**	30 (100.0)
** No**	0 (0.0)
**Insurance status**	
** Medicare**	17 (56.7)
** Medicaid**	0 (0.0)
** Medicare & Medicaid**	9 (30.0)
** More than one**	4 (13.3)
**Walking aids^b^**	
** Yes**	13 (43.3)
** No**	17 (56.7)

a Examples of professional degrees include JD (Juris Doctor) and MD (Doctor of Medicine).

b Walking aids were recorded as either a cane or walker, irrespective of their specific type or brand.

The exit interviews yielded data on how older adults with MCI perceived the passive monitoring device. Based on the a priori categories, several subcategories emerged from the data (Figure 3 and Table S4).

**Figure 3.**
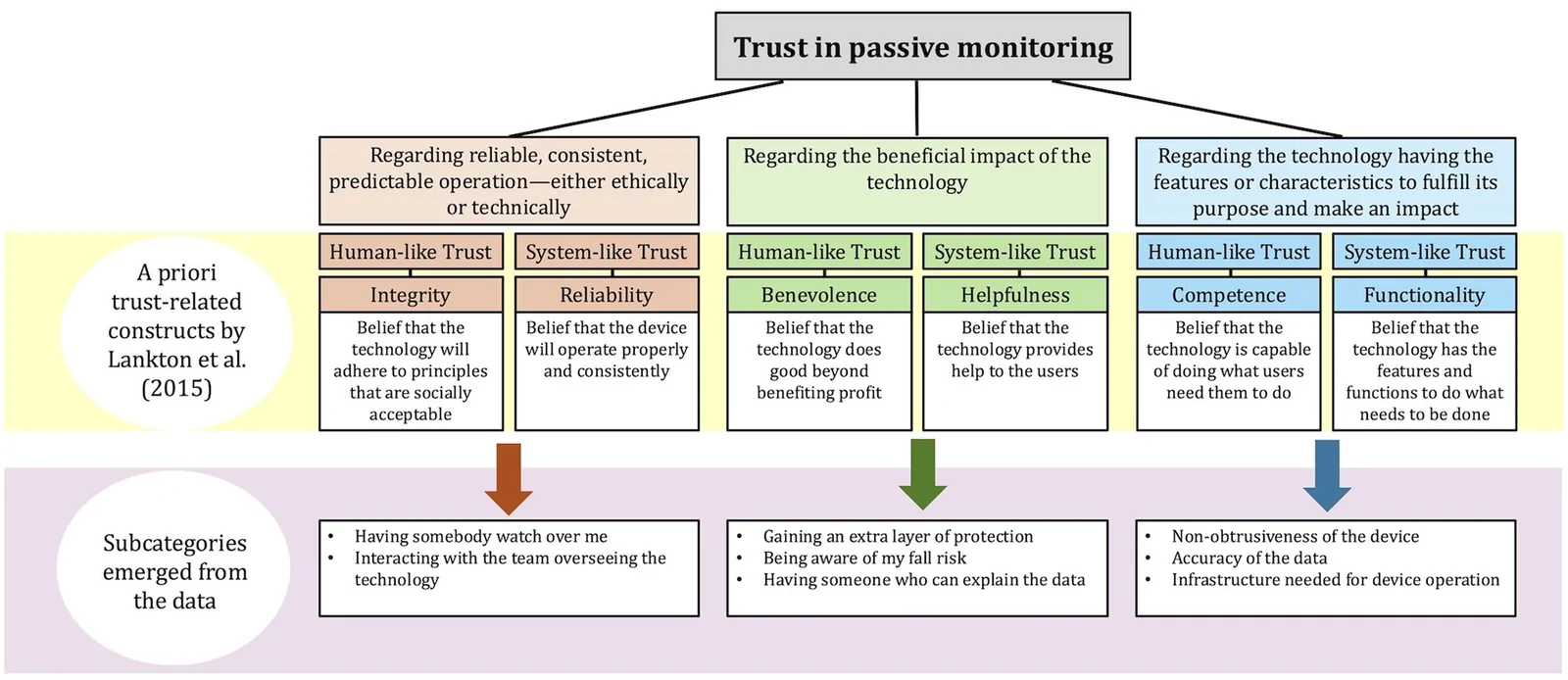
Predetermined trust-related constructs with subcategories that emerged from the data.

### Integrity and reliability

The first pair of trust-related constructs is related to consistent, dependable, and predictable operation, whether ethically or technically. Under these constructs, two subcategories emerged.

#### Having somebody watch over me

In this subcategory, participants described the sensor’s omnipresence and thoughts related to privacy. Participants perceived the depth sensors not only as a technical device, but as a means for other people to watch them. The large majority reported being positive towards using technology to track gait information. Some participants explicitly commented that they were glad to be monitored and felt secure knowing they were being watched: (“*I felt more secure, you know, because it seems like it was somebody watching over me*” [G0001, Female, 70]). However, many added that they had initial concerns regarding situations where their bodies might be exposed: “*I didn’t have any problems with it. Sometimes I was afraid to come out here in a certain way*” (G0051, Female, 84). Understanding that the depth sensor captured their silhouette and not actual images relieved such worries for some. Others, however, took precautionary actions, such as avoiding walking around with no clothes or wearing thick coats in the home. While such privacy-related concerns were commonly discussed, only two explicitly used the term “*privacy*” when reflecting on the impact of being monitored. One described feeling ambivalent about omnipresent technology (“*I know there are benefits to it, but I guess, for me, it steps on your privacy a little*” [G0060, Male, 73]), whereas the other participant had the opposite view (“*I would recommend it to anyone who was willing to, how would I say, be monitored because it’s not any intrusion, I don’t think it is, any intrusion on our privacy*” [G0013, Female, 78]).

The large majority responded that they did not have any concerns about how the data were collected or how the device operated. There was one 78-year-old female (G0013) participant who mentioned having concerns only when she heard “*funny things on TV*” related to people spying on others through artificial intelligence or technology. However, she did not request to have the device removed or have it shut off, telling us that she was accustomed to using technology. In some cases, it was the friends, families, or other acquaintances who were more concerned about the device, with the participants having to explain what it was for: “*A lot of them was like, is it recording me? And I’m like, no, it ain’t paying you no attention. Just keep doing what you’re doing*” (G0009, Female, 65).

#### Interacting with the team overseeing the technology

In this subcategory, participants described aspects of the research team’s conduct and data stewardship that made them feel safe throughout their experience with the sensor. Initial meetings played an important role in establishing early trust: “*I made up my mind from the beginning based on your conversation. When you first talked to us about it that I felt safe in doing it*” (G0035, Female, 69). Participants were also reassured by directly seeing how the images would appear on screen: “*You showed me all I needed to know when you showed me what appears on the monitor*” (G0056, Female, 69). One participant commented that seeing our team member install the device led her to feel secure, while another added that our team not asking personal questions led her to be feel unconcerned about data collection and device operation. Moreover, knowing that other third parties would not have access to their data contributed to their trust. Another commented that, given the community’s concern about constant monitoring, the team must maintain ongoing interactions and follow-ups to successfully implement the technology: “*The follow-up is, you know, I think is mandatory. You can’t do this without coming back and talking with us*” (G0035, Female, 69).

### Helpfulness and benevolence

The second pair of trust-related constructs relates to the beneficial impact of the technology perceived by the users. Three subcategories emerged under these constructs.

#### Gaining an extra layer of protection

In this subcategory, participants discussed how the device would be helpful for those with older age and higher fall risks. Many perceived falls to be a serious problem for older adults: “*I do believe it will help because in the past I actually did have some falling issues due to health reasons. And I really am very mindful of falling because I know for a senior, it’s really serious. Could be a step to fall down and then go to the hospital and then you never come home*” (G1086, Female, 67). One participant added that living alone made them have to watch out for themselves; another commented that older adults may not be aware of their fall risks: “*I think it’s a good thing, because there’s some people that don’t even know they have an unsteady gait. Until somebody show them or tell them, let them know about it*” (G0039, Female, 67). Being aware of the device’s presence constantly reminded some participants to be more careful and to exercise more. One participant reported that she did not want others to see her fall through the device recording, which led her to be extra cautious when walking.

Participants reported feeling safer with the device, seeing the device as a way for others to capture early signs of falls and to help prevent them: “*I had eyes on me, you know, to help me*” (G0016, Female, 70). Similar sentiments were shared through descriptions such as a “*security blanket*” (G0003, Female, 80), “*medic alert*” (G0001, Female, 70), and “*an extra safety net*” (G0056, Female, 69). Two female participants described the device as “*an extra layer of protection*,” with one adding that the depth sensor is akin to an implanted medical device: “*especially if you have already a device on the body, that’s two people definitely looking out for you. Making sure you’re okay*” (G1067, Female, 70).

Five participants told us that the device would be helpful when falls actually occurred: “*It did feel good that if I was to fall and was laying there that someone would know this*” (G0056, Female, 69). Among them, two participants shared stories of their neighbors who had fallen and were found too late, highlighting that the device would have been helpful during those crises. One participant added that having the device made her realize she would not be alone in an event of a fall. Two other participants mentioned alternative devices, such as call bells or smart watches, but commented that these may not be as helpful when they fall because they might be unable to reach the call bell or might not be wearing their watches. However, one participant argued that she did not see the depth sensor being critical to her safety unless “*there’s some way of communicating to whoever’s monitoring it, and I doubt if it’s being monitored 24-7*” (G0013, Female, 78).

There were a minority of participants who reported benefits of the depth sensor beyond its original intent of identifying fall risks. Two participants, with one sharing a story of her neighbor experiencing an unwanted intrusion in her household, deliberately let their neighbors mistake the depth sensor for a security camera: “*Cause the way it looks, it looks like a pure camera. And it looks like I have a camera in here in case anybody come in here and think that’s a real camera. I have a camera. And that’s what I did. I leave it as that*” (G0016, Female, 70). Additionally, the 78-year-old female participant (G0013) who questioned the depth sensors’ benefits for safety reported it was actually beneficial for social interactions. The device served as a “*great conversational topic*” that gave her opportunities to actively engage with others in the community.

#### Being aware of my fall risk

In this subcategory, participants discussed about the benefits of the resulting data and the dashboard. Many reported that the data would help them be aware of their fall risk. One participant described the dashboard as a factual reference for their current gait: “*Yeah, made me more aware of the reality, you know, instead of what I’ve convinced myself or what I think I do*” (G0055, Male, 72). Beyond factual information, two participants reported that the data offered comfort: one found that the data showed they were doing better than they had perceived, while another said reviewing the data made her more comfortable with her onset disability. Some commented that the data would serve as a foundation for seeking timely interventions or feedback to improve their gait. One participant commented that, if they do fall, the data could serve as a source for understanding why. However, three participants commented that they would not be able to change their gait even if the data or display were given to them, which limited the perceived helpfulness of the generated information.

The large majority reported that they were willing to show their data to their doctors, nurse practitioners, physical therapists, and family members: “*It helps people or doctors to know, you know, what’s going on in the home and can figure out medical issues also*” (G1085, Female, 71). Participants described that sharing the data would help others understand what they are doing in their household, what issues are happening in the home, and how at risk they are. Yet, one participant pointed out that others would have to first understand the device’s purpose and how the program operates, rather than simply having the data shared with them.

#### Having someone who can explain the data

Eight participants commented that they needed someone to explain and interpret the dashboard to find the data personally helpful: “*Actually, for the average person like myself, I appreciate you going over with me, but if I looked at this, I really wouldn’t know what it was. Even though I have a college degree, I kind of forget how to read these types of maps*” (G1086, Female, 67). Three other participants described the importance of ongoing communication to provide feedback about their gait and answer questions. Related to this, one participant described our research member in the field as “*the catalyst for this*.”

### Functionality and competence

The third pair of trust-related constructs represents the belief that the technology has the features, characteristics, and capability to fulfill its purpose. Under these constructs, we identified three subcategories related to the device’s purpose of nonobtrusively capturing older adults’ gait patterns.

#### Non-obtrusiveness of the device

In this subcategory, participants discussed the depth sensor’s obtrusiveness in their daily life. The large majority reported not noticing the device: “*It was good. It was like it wasn’t even there. It didn’t bother me at all*” (G0022, Female, 69). Even participants who initially had concerns about being monitored told us that they eventually forgot that the device was there. Four participants reported that, every now and then, they would intentionally check on the device to see whether it was working: “*Most of the time I wasn’t paying attention to it, except that I sit directly in front of the camera and I like to watch the light speaking*” (G0013, Female, 78). All participants mentioned that its presence did not interfere with their home environment. The large majority also reported that the device did not affect their sense of independence: “*I just kept doing what I usually do, where I usually go*” (G0020, Female, 86). When it did impact their independence, participants described only positive impacts, such as feeling more secure or trying to walk more carefully. The majority reported that the device did not interfere with daily routines; when they did, the changes were mostly about trying not to be undressed in front of the device.

#### Accuracy in system-generated gait information

In this subcategory, participants discussed how accurately the data reflected their gait patterns. Most participants replied that the data seemed accurate. From the display, some recalled specific events that may have changed their walking pattern, such as using a walker or their home temperature affecting their walking speed. One participant added that data accuracy was important for tolerating constant monitoring: “*As long as it’s accurate at recording what it’s supposed to do, it does its job. It don’t bother me*” (G1061, Female, 73). Two discussed false positives: One participant expressed concerns of the device misinterpreting aggressive or sudden movements as falls or confusing the movements of pets with theirs. The other participant described an event where their grandson jumped in front of the device, which triggered a couple of fall notifications: “*My grandson said, ‘I just wanted to see what’s going to happen if they’re going to call you again*’” (G1067, Female, 70). However, there were six participants who could not confirm the accuracy of the data. Three responded that they did not know what to expect regarding their gait: “*Well, it is what it is. It’s seeing how I walk, my strides, my steps or whatever. I don’t know because I really never pay attention to how I walk*” (G0051, Female, 84). The other three could not easily answer because they could not interpret the data: “*Because I, I don’t know what this means and that means*” (G1030, Female, 77).

#### Infrastructure and indicators of proper functioning

In this subcategory, participants discussed conditions under which they believed the device would properly function and indicators they relied on to determine whether it was working. There were instances where participants questioned the device’s operation, such as when in the dark or when temporarily lacking necessary services like electricity. In such cases, flashing red lights served as indicators for proper functioning: “*I noticed the light flashing, so I guess it did [see me]*” (G0013, Female, 78). One participant was concerned that the device would not work when she could not pay her telephone bill, not realizing that the device used internet connectivity instead of phone services. Additionally, one participant expressed concerns over people needing Wi-fi connection for the device to function: “*Wi-fi issue may not necessarily work for everybody. So, it’s good for me because I have it, but there are people here who need to have this, but they can’t*” (G0035, Female, 69).

## Discussion

We found our participants’ conceptualization of trust in passive monitoring to be complex, as they perceived the technology both as a technological system and as a proxy for other people overseeing their daily activities. For constructs related to dependable and predictable operation (integrity and reliability), participants’ descriptions aligned more with human-like trust, as they associated depth sensors with human agents monitoring them. They frequently shared thoughts on comfort and privacy aspects of having sensors in their homes. Conversely, within other paired constructs (helpfulness and benevolence; functionality and competence), our participants’ narratives aligned more with system-like trust, focusing on the device’s utility and seamless operation. These mixed findings are notable in light of Lankton et al.’s^15^ framework, as the authors suggested that trust constructs employed in empirical studies should align with the technology’s “humanness”—the degree to which the technology possesses human-like qualities (eg, voice or interactive features). However, our findings suggest that, even though the hardware of passive sensors is itself designed to operate unobtrusively and does not have any characteristics resembling humans, the distinction between human- and system-like trust constructs was not straightforward and clear-cut. Lie et al.,^14^ who more broadly focused on ambient home monitoring systems for older adults, similarly found that the institutions overseeing the technology and its data played an important role in bolstering participants’ trust. In their study, participants placed greater trust in the technology when offered by universities or public entities than private companies.^14^ In our study, older adults with MCI did not comment on organizational types. Yet, they described specific actions by the overseeing team that made them feel safe, such as having individual conversations prior to device setup, demonstrating how the images would appear, and ensuring that the data were not accessible to third parties. Taken together, these findings suggest that even though passive monitoring technology does not involve active data collection and device operation by humans, older adults’ perceptions of the team behind the technology must be considered in its design and community deployment.

We also found that having someone actively interpret the data and maintain ongoing responsiveness to the system-generated information added a crucial layer to older adults’ trust in passive monitoring systems. Despite the depth sensor’s passive nature that does not require end users’ active engagement, participants highlighted the importance of receiving personalized explanations of their data and responses to device-related questions. Such findings align with prior work showing that participants expected feedback from the team behind passive technologies to help act on their data, considering this feedback important for sustained engagement.^32^ These findings altogether suggest that human operators’ responsiveness to the data and provision of ongoing feedback were critical in participants’ understanding the system’s logic and to promoting its perceived helpfulness and functionality, ultimately fostering trust.

Our participants confirmed the inherent unobtrusiveness of the device, which captured their naturalistic activities without interfering with their daily routine.^33^ Our participants also found the device helpful in many ways. A commonly reported benefit was having additional set of “*eyes*” that could capture early signs of a fall. Living alone without anyone to check on them or having a history of falls reinforced the perceived helpfulness of monitoring devices. Some even shared stories in which neighbors were not found promptly after falling and commented that the device would have been helpful in such situations. Additionally, being aware of their walking pattern was perceived to be mentally reassuring by some, either because it could serve as a preventive measure or because it encouraged them by showing that they were doing better than they thought. Simply having a depth sensor installed in their household sometimes added a sense of physical safety or even facilitated enjoyable social engagement. Compared to studies exploring the impact passive sensors have on older adults’ actual physical health, very few studies have explored the impact the technology has on other outcomes, such as emotional security or mental health.^34^ Our findings highlight that passive monitoring may benefit older adults with MCI beyond physical safety, but the effectiveness of these broader outcomes requires further research.

Another noteworthy finding involved our participants’ perceptions of privacy. Many participants reported having initial privacy-related concerns, especially during moments when they were undressed in their homes. However, when asked if they had any concerns regarding how the data were collected, most replied that they did not. Privacy concerns were indeed quickly resolved by recognizing that the device captures their appearances as silhouettes, similar to the findings of Berridge.^35^ Yet, at a deeper level, our participants may have conceptualized daily monitoring and data collection as separate processes. For instance, the 73-year-old male participant who commented that technology tracking real-time walking patterns felt as if “*it steps on your privacy a little*” also stated that he had no concerns about how the data were collected or how the device operated. Although the idea of being observed in intimate situations was seen as uncomfortable, our participants may have placed implicit trust in the data collection process and believed that the technology is serving its purpose. Such findings add to the literature that privacy is indeed not a homogenous concept, differentiating physical privacy (ie, related to the degree of one’s physical territory, including their home, personal space, or body, being seen) from informational privacy (ie, related to access, protection, and sharing of one’s personal information).^36^^,^^37^ Birchley et al.^38^ reported that engineers designing smart home health technology tend to focus more on informational privacy than on physical privacy. To promote trust and acceptability of the technology, it is worth further exploring how older adults with MCI weigh privacy-related concerns in relation to passive monitoring devices.

Our findings have implications for future research and technology adoption for community-dwelling older adults with MCI. First, our findings highlight that institutions or organizations overseeing the technology should first establish trusting relationships with community members before implementing passive monitoring within the households of older adults with MCI. As implied by one of our participants’ quotes (“*You can’t do this without coming back and talking with us*”), ongoing interactions with the target population is critical. In this regard, research utilizing participatory design to involve older adults as trusted partners would be helpful.^39^ Second, despite the unobtrusive nature of passive monitoring, our findings highlight that passive monitoring does not eliminate the need for human involvement. Participants appreciated having someone to ask questions, receive advice, or request for help, which was associated with promoting the technology’s perceived helpfulness and functionality. These results suggest that effective passive monitoring programs must integrate both technological and relational components to build trust among older adults with MCI. Finally, our findings highlight that having the device encouraged our participants to proactively manage their safety by walking more carefully or engaging in exercise. Future research is needed to examine whether such sense of empowerment could contribute to long-lasting behavioral changes and fall prevention.^13^

This study has several limitations. First, we were only able to interview those who were willing to participate in research and install a depth sensor in their residence for a year. Hence, we may have missed participants who declined to participate because they did not trust the device or had significant concerns about data privacy. Also, recall bias may have been introduced as the interviews were conducted once participants completed the study. Future studies utilizing multiple time points for qualitative data collection could capture real-time perceptions of trust and acceptability and explore how these perceptions evolve over the study period. Additionally, our setting was limited to one geographical location within the eastern United States and the majority of our samples were Black females. Although data redundancy was achieved, our findings may not be transferable to other settings with different sociocultural norms regarding the use of monitoring technology.

## Conclusions

Our findings contribute to understanding how trust in technology manifests among older adults with MCI, a population previously underexplored in trust-related research. Participants viewed the passive monitoring device through a dual lens: as both a technological tool and as a proxy for human agents monitoring their daily activities. Despite the unobtrusive nature of depth sensors, results indicate that trust depends on establishing sustained relationships with oversight organizations and continuous involvement of human coaches who can facilitate communication and promote system explainability. Future research should prioritize participatory design methodologies that amplify the perspectives of older adults with MCI in creating human-centered, trustworthy passive sensing technologies for comfortable home use and effective fall prevention.

## Supplementary Material

glag018_Supplementary_Data

## Data Availability

De-identified transcripts may be available upon request.
