# Supplementary material for: “I had eyes on me to help me”: a qualitative descriptive study on trust in passive monitoring systems among older adults with mild cognitive impairment
Source: J Gerontol A Biol Sci Med Sci. 2026 Jan 30;81(3):glag018. doi: 10.1093/gerona/glag018 (PMC12967069; doi:10.1093/gerona/glag018)
Supplement: Supplementary file 1 [file glag018_supplementary_data.docx]

Supplementary Table 1: Consolidated criteria for reporting qualitative studies (COREQ): 32-item checklist^1^

| **Item No** | **Guide Questions/Description** | **Location** |
| --- | --- | --- |
| **Domain 1: Research team and reflexivity** | | |
| **Personal Characteristics** | | |
| 1. Interviewer/ facilitator | Which author/s conducted the interview or focus group?   - Second author (SH) | Settings and procedure; Fourth paragraph |
| 2. Credentials | What were the researcher’s credentials? E.g., PhD, MD   - Sean Harrison, MPH, CPT | Title page |
| 3. Occupation | What was their occupation at the time of the study?   - Senior Research Coordinator | Supplementary Table 3 |
| 4. Gender | Was the researcher male or female?   - Male | Supplementary Table 3 |
| 5. Experience and training | What experience or training did the researcher have?   - Sean Harrison is a masters-level public health practitioner pursuing a PhD in exercise physiology. His professional experiences in personal training, pharmacy benefit management, and research coordination contributed to his overall toolkit. Sean has experience conducting qualitative data collection, having led interviews with older adults in prior projects (e.g., R03AG069793, R49CE003083). | Supplementary Table 3 |
| **Relationship with participants** | | |
| 6. Relationship established | Was a relationship established prior to study commencement?   - Sean Harrison did not know study participants prior to their enrollment and no prior relationship was established. | Supplementary Table 3 |
| 7. Participant knowledge of the interviewer | What did the participants know about the researcher? e.g. personal goals, reasons for doing the research?   - Participants were advised the coordinator was staff/researcher from the University conducting the study for a university faculty member. Each participant was advised of this prior to enrollment; however, some reverted to their own belief that this research was for a dissertation. | Supplementary Table 3 |
| 8. Interviewer characteristics | What characteristics were reported about the interviewer/facilitator? e.g. Bias, assumptions, reasons and interests in the research topic   - Sean is a PhD student whose expertise in exercise and public health, and research experience contributes to the success of older adult research investigations. | Supplementary Table 3 |
| **Domain 2: study design** | | |
| **Theoretical framework** | | |
| 9. Methodological orientation and Theory | What methodological orientation was stated to underpin the study? e.g. grounded theory, discourse analysis, ethnography, phenomenology, content analysis   - Content analysis | Study design |
| **Participant selection** | | |
| 10. Sampling | How were participants selected? e.g., purposive, convenience, consecutive, snowball   - Convenience sampling | Setting and procedures; First paragraph |
| 11. Method of approach | How were participants approached? e.g., face-to-face, telephone, mail, email   - Face-to-face | Setting and procedures; First paragraph |
| 12. Sample size | How many participants were in the study?   - The dataset consisted of interviews from a sample of 30 participants randomly selected from the total pool of 67 who completed the study | Data analysis; First paragraph |
| 13. Non-participation Setting | How many people refused to participate or dropped out? Reasons?   - A total of 78 older adults with MCI enrolled, of whom 67 completed the entirety of the study. Two expired and nine withdrew due to various reasons (e.g., installation, internet, or other technical issues, physical inactivity, and personal reasons unrelated to the study). | Setting and procedures; fourth paragraph |
| 14. Setting of data collection | Where was the data collected? e.g., home, clinic, workplace   - Home | Setting and procedures; fourth paragraph |
| 15. Presence of nonparticipants | Was anyone else present besides the participants and researchers?   - Individual interviews were conducted | Setting and procedures; fourth paragraph |
| 16. Description of sample | What are the important characteristics of the sample? e.g. demographic data, date   - Table 1 | Table 1 |
| **Data collection** |  |  |
| 17. Interview guide | Were questions, prompts, and guides provided by the authors? Was it pilot tested?   - Supplementary Table 2; Yes, it was pilot-tested with five older adults to confirm clarity and comprehensibility. | Supplementary Table 2,  Setting and procedures; fourth paragraph |
| 18. Repeat interviews | Were repeat interviews carried out? If yes, how many?   - No. Interviews were conducted once per participant. | Settings and procedure; fourth paragraph |
| 19. Audio/visual recording | Did the research use audio or visual recording to collect the data?   - All interviews were audio-recorded | Settings and procedure; fourth paragraph |
| 20. Field notes | Were field notes made during and/or after the interview or focus group?   - No | Settings and procedure; fourth paragraph |
| 21. Duration | What was the duration of the interviews or focus group?   - Approximately 30 minutes | Settings and procedure; fourth paragraph |
| 22. Data saturation | Was data saturation discussed?   - After coding 30 transcripts in total, three additional randomly selected transcripts were assessed to confirm data redundancy. New codes or ideas did not emerge from these additional transcripts, confirming that data redundancy was achieved. | Data analysis; second paragraph |
| 23. Transcripts returned | Were transcripts returned to participants for comment and/or correction?   - No | Data analysis; first paragraph |
| **Domain 3: analysis and findings** | | |
| **Data analysis** | | |
| 24. Number of data coders | How many data coders coded the data?   - Two authors (OO and GD) independently coded 10 transcripts and discussed until reaching consensus. Subsequently, the first author coded the remaining 20 transcripts. | Data analysis; second paragraph |
| 25. Description of the coding tree | Did the authors provide a description of the coding tree?   - Figure 3 | Figure 3 |
| 26. Derivation of themes | Were themes identified in advance or derived from the data?   - Findings are presented under the model’s constructs. | Abstract; Results |
| 27. Software | What software, if applicable, was used to manage the data?   - Atlas.ti software | Data analysis; first paragraph |
| 28. Participant checking | Did participants provide feedback on the findings?   - No. Member checking was not used as a method to ensure the findings’ rigor. | Data analysis; third paragraph |
| **Reporting** | | |
| 29. Quotations presented | Were participant quotations presented to illustrate the themes/findings? Was each quotation identified? e.g., participant number   - Yes | Throughout the results section |
| 30. Data and findings consistent | Was there consistency between the data presented and the findings?   - Yes | Throughout the results section |
| 31. Clarity of major themes | Were major themes clearly presented in the findings?   - Yes | Figure 3 |
| 32. Clarity of minor themes | Is there a description of diverse cases or a discussion of minor themes?   - Yes | Throughout the results section |

1.	Tong A, Sainsbury P, Craig J. Consolidated criteria for reporting qualitative research (COREQ): a 32-item checklist for interviews and focus groups. *Int J Qual Health Care*. 2007;19(6):349-357. doi:10.1093/intqhc/mzm042

Supplementary Table 2: Interview guide

Introduction

I would like to ask you some questions so we can better understand your experience in the study and how to refine our intervention. Is it OK with you if I record our conversation? This recording will be kept confidential and only used for our analysis; you will not be identified or linked to any of your responses.

General Questions

• How was your overall experience with this sensor in your home?

• What do you think about the use of technology to track information about how people walk?

• What about your daily routine did you change or adjust because of the depth sensor?

• What were the challenges or issues you experienced?

• Did you feel this device/ sensor may have interfered with your home environment or personal

space?

• Did the device impact your interactions with others? (Did visitors notice or ask you about it?)

• Did the sensor impact your sense of independence?

o Please elaborate

Sensor-related Questions

• How might having a sensor like this be useful to yourself or someone who may be a fall risk or showing signs of fall-risk?

• Do you have any concerns about how the data is collected or the device operates? If so, please elaborate.

Dashboard Questions

Next I will show you the dashboard data that has been collected from your walks. (Explain the graphs).

• What are your thoughts on the graphs shown?

a. What do you find interesting about the displays?

b. What do you find confusing about the displays?

c. How accurately do you think the data reflects your activity and well-being?

d. Are the displays helpful in anyway?

- How might you use these displays, if at all?

e. How frequently would you look at this information?

• What suggestions would you have for improving the displays?

• What information on the visual display would be helpful to you as feedback?

• Are there alternative ways in which you would like the sensor information to be presented?

• How do you think your health/wellbeing might change if you received the data regularly?

• If you had access to this information on a regular basis, how would you like it delivered? (e.g., paper copy with graphics, access via smart phone, semi-regular discussions with healthcare professional)

• Would this information be useful to share with others? (e.g., loved-one’s/caretaker, physical therapist, physician)

o With whom would you share the information?

• Do you have any further comments to make about the sensor data and the graphs?

Supplementary Table 3. Reflexivity statement

As the lead author of this qualitative descriptive study, I (**Oonjee Oh**) enter this work with a deep commitment to ensuring that the perspectives and voices of older adults are integrated into the design and implementation of technologies intended for them. I am a female PhD candidate in the School of Nursing, and I bring both my clinical background as a registered nurse and research experience in qualitative methods. I am also aware that my previous experiences with older adults who faced challenges with understanding and utilizing technology may impact how I interpret the narratives of the study samples.

Interviews for this qualitative study were conducted by **Sean Harrison,** a male PhD student in in exercise physiology whose expertise lies in exercise and public health. His research program contributes to the success of older adult research investigations. As a master’s-level public health practitioner, Sean has experience conducting qualitative data collection, having led interviews with older adults in prior projects (e.g., R03AG069793, R49CE003083). Sean served as a senior research coordinator during the study, and he did not know any study participants before enrollment. Sean’s professional experiences in personal training, pharmacy benefit management, and research coordination contributed to his overall toolkit. Sean reviewed the findings and the resulting reports and provided feedback.

With the aim of mitigating potential biases, I independently coded subsets of the study transcripts with **Dr. George Demiris**, an informatics researcher and gerontologist. Findings and iterations of the final report were shared with **Drs. Justine Sefcik**, **Therese S. Richmond, and Nancy Hodgson,** who collectively bring expertise in qualitative methods, nursing science, nursing-driven interventions, and dementia research. Throughout the process of investigator triangulation with Dr. George Demiris and peer debriefing with the entire research group, my internal biases were challenged, and we ensured that the findings were firmly grounded in the participants’ quotes rather than my own perspectives.

Supplementary Table 4. Categories, subcategories, and codes

| Pre-determined trust-related categories | Subcategories identified from the data | Example quotes |
| --- | --- | --- |
| Pair I  *Integrity & Reliability* | Having somebody watch over me | - Sense of security: “*I really liked it because it felt like somebody was there like security. Right. It was watching me. I knew that it was watching me, so I really liked it (G0043)*” - Concerns: *“At first, I was kind of like, hmm, spying on people? You know, that's my first impression. Girl, you want that, then they're going to be spying on you. (G0030)”*   - Moments of nudity/minimal clothing: *“I've been fine and comfortable with it. In the beginning I was afraid to run around naked, but other than that, not that I do that anyway (G0013)”*   - Relieved by silhouette-based images: “*just a silhouette. Couldn't tell if it was anything particular about me. (G1030)”*   - Taking precautionary actions: “*I am a grandmother, I have to, I was cognizant of it being there, so to be honest with you, I always made sure I had something on when I came into the room. (G0035)*” - Friends, families, or other people were more concerned: *“I have neighbors of mine who think I'm crazy for having that in there…. they shunned me and said, why would you want that? Why would you want somebody to be looking? I said, you're not understanding. It's not somebody who's looking. (G0035)”* |
|  | Interacting with the team overseeing the technology | - Initial meeting: *“I made up my mind from the beginning based on your conversation. When you first talked to us about it that I felt safe in doing it. (G0035)”* - Seeing images generated by the device: *“you showed me all I needed to know when you showed me what appears on the monitor. (G0056)”* - Observing device installment: *“everything was fine. I saw you when you installed it, so I was pretty secure. (G0043)”* - Third-party data access: *“No, because this is going straight into your location in the office where you can monitor it (G0051)”* - Ongoing follow-ups with the team: *“The follow-up is, you know, I think is mandatory. You can't do this without coming back and talking with us (G0035)”* |
| Pair II  *Helpfulness & Benevolence* | Gaining an extra layer of protection | - Falls perceived as a serious problem for older adults: *“I'm all for it because that is, you can fall in your knees and without even breaking them you can cause like several months’ worth of physical therapy to happen. (G0056)”* - Living alone: *“If you do say I'm still at a kind of fall risk, I know I have to do a little bit more to protect myself, too. Because I don't have nobody watching me, but I'd have to watch myself, right? (G0020)”* - Might not know their fall risk: *“I think it's a good thing, because there's some people that don't even know they have an unsteady gait. Until somebody show them or tell them, let them know about it. (G0039)”* - Being aware of the device constantly reminded them to be more careful: *“So, it made me obvious that I was trying to walk better (G1009)”* - Did not want others to see them fall: *“I said oh somebody's gonna see me if I fall, I don't want to see me falling then again I don't want to fall (G0022)”* - Others can capture early signs of falls: *“All I know that I had eyes on me, so I had eyes on me, you know, to help me, you know, because sometimes I will make a mistake and trip, you know what I mean, but never fall. But it's good that it's something good that y'all can catch, y'all might need to, you know, help me to prevent (G0016)”* - Similar to medical device: *“It definitely gives you an extra layer of protection, especially if you have already a device on the body. That's two people definitely looking out for you. Making sure you're okay. (G1067)”* - Helpful during crisis: *“It did feel good that if I was to fall and was laying there that someone would know this (G0056)”*   - Neighbors found too late: *“One of the guys around here I think in 411, 413 he recently passed away. He fell and hit his head. Oh no. And they found him in the apartment a couple days later. I don't know if you ever come in and you may have seen he there's two guys that sit outside and smoke. The Hispanic guy is the one who fell and hit his head. And see, if he had had this device in his apartment, maybe somebody would have seen it and he would have gotten help faster. (G0022)”*   - Not alone: *“It also made me realize that if I was to fall, I wouldn't be by myself. (G0022)”*   - Other devices: *“I think the device would be very useful because we have all these call bells. Who's to say if you fall in the middle of the living room you're going to be able to get over to the call bell? Secondly, these call bells go nowhere. You ring the call bell nothing happens (G0056)”*   - Having channels for communication being important: *“I really don't think it's helpful at all unless there's some way of communicating to whoever's monitoring it, and I doubt if it's being monitored 24-7. (G0013)”* - Device being helpful beyond its original intent:   - Preventing unwanted intrusion: *“Cause the way it looks, it looks like a pure camera. And it looks like I have a camera in here in case anybody come in here and think that's a real camera. I have a camera. And that's what I did. I leave it as that. That's what y'all think. I know what it is. (G0016)”*   - Facilitating social engagement: *“It does give a great conversational topic. It really does. (G0013)”* |
|  | Being aware of my fall risk | - Gait data as factual reference: *“Yeah, made me more aware of the reality, you know, instead of what I've convinced myself or what I think I do. (G0055)”*   - Doing better than they thought: *“So mentally, you know, it, from what you showed me and explained to me, it's making me feel mentally better that I might be at average or just point a little bit above average. So that's a good mental thing for me because sometimes the way I'm feeling and walking around thinking, oh, is I going to fall? Like giving into it, but see, that's a booster. That's a mental booster to me that it could be worse. (G1061)”*   - Increased comfort regarding disability onset: *“I think that it has made me become more comfortable with my onset disability. (G0035)”*   - Supporting timely interventions: *“Well I would ask someone for some type of program that would help me improve it or alter it or I'm going to say actually stop it. You know, like if you do this, this will help you. Not do that. (G1030)”*   - Understanding reasons for a fall: *“In case they do have a fall, you can pretty much, you know, figure out why. (G0045)”*   - Can’t change my stride: *“I don't see how the length of my stride or the speed of my stride affects me in any way. Or this information. If it's given to me, there's nothing I can do with it. (G0013)”* - Willing to share data with others: *“I know my daughter would love to know about this. Okay. I mean it really makes sense. I don't think about it too much because I'm still under 70, but I know in 10 years from now or maybe even less it really would be helpful for me to have this. And I think my daughter would feel even more secure that I'm doing good. (G1086)”* |
|  | Having someone who can explain the data | - Need someone to explain and interpret the data: *“Actually, for the average person like myself, I appreciate you going over with me, but if I looked at this, I really wouldn't know what it was. Even though I have a college degree, I kind of forget how to read these types of maps (G1086)”* - Need ongoing feedback: *“You can't do this without coming back and talking with us. (G0035)”* |
| Pair III  *Functionality & Competence* | Non-obtrusiveness of the device | - Did not notice the device: *“Actually it's been non-intrusive. I've been fine and comfortable with it. (G0013)”*   - Sometimes check on the device to see if it is working: *“I didn't even really know it was there. No? You know, every once in a while, I sit down on my sofa, I would look up and I'd see it. I'd wave at it a couple of times and see the little red light going around inside. But other than that, I really didn't pay any attention to it. (G0060)”* - Sense of independence: *“No. I still did me. (G0039)”* - Daily routine:   - No change: *“I didn't change my routine. I went about doing things that I always do (G0017)”*   - Changes: *“There were times when I remembered that the camera was there and said, you can't do that, the camera's there. But after a while, I forgot and found myself doing stuff that I normally wouldn't do, but it was already done. (G0003)* - Interactions with others:   - No change: *“No, no. People that came in, I don't think they even noticed it was there because they never said anything. (G0001)”*   - Changes: *“The only one who had a problem was my son. He's a very skeptic. You know? Mom, be careful how you walk around here. Be careful what you say. Be careful what you do. You know, your conversations with your friends. (G0045)”* |
|  | Accuracy in system-generated gait information | - Data seemed accurate: *“I think it's accurate. I do. I think it accurately reflects it. (G0035)”*   - Specific events: *“It looks accurate to me because, like I say, I think that I would have been walking faster in January because it was very cold in February. So it's pretty accurate. (G0043)”* - Willing to tolerate constant monitoring given its accuracy: *“As long as it's accurate at recording what it's supposed to do, it does its job. It don't bother me. (G1061)”* - Concerns about false positives: *“That was the bigger one, and the little one likes to jump off of everything. So, he jumped, so they both, I think they just were trying to test it. You know, and I'm like, ‘Don't jump’. And my grandson said, ‘I just want to see what's going to happen if they're going to call you again.’ (G1067)”* - Hard to confirm data accuracy: *“Well, it is what it is. It's seeing how I walk, my strides, my steps or whatever. I don't know because I really never pay attention to how I walk. (G0051)”* - Need to be able to read the data to confirm its accuracy: *“I couldn't tell you that. Because I, I don’t know what this means and that means (G1030)”* |
|  | Infrastructure and indicators for proper functioning | - Electricity: *“I just wondered sometimes if I ran around in the dark, if it saw me, but I noticed the light flashing, so I guess it did. (G0013)”* - Wi-Fi: *“Wi-fi issue may not necessarily work for everybody. So, it's good for me because I have it, but there are people here who need to have this, but they can't (G0035)”* - Phone services: *“I was concerned when my telephone got cut off because I didn't pay the bill as to whether or not it was still working. (G0003)”* |
